# A Study of the Gut Bacterial Community of *Reticulitermes virginicus* Exposed to Chitosan Treatment

**DOI:** 10.3390/insects11100681

**Published:** 2020-10-08

**Authors:** Telmah Telmadarrehei, Juliet D. Tang, Olanrewaju Raji, Amir Rezazadeh, Lakshmi Narayanan, Rubin Shmulsky, Dragica Jeremic

**Affiliations:** 1 Department of Sustainable Bioproducts, Mississippi State University, 201 Locksley Way, Starkville, MS 39759, USA; lanraj@gmail.com (O.R.); lakshmi.anarayanan@gmail.com (L.N.); rs26@msstate.edu (R.S.); dragica.jn@gmail.com (D.J.); 2 Forest Products Laboratory, USDA Forest Service, 201 Lincoln Green, Starkville, MS 39759, USA; juliet.d.tang@usda.gov; 3 Department of Chemical Engineering and Applied Science, University of Toronto, 200 College Street, Toronto, ON M5S 3E5, Canada; 4 Institute of Food and Agriculture Science, University of Florida, 8400 Picos Road, Ste. 101, Fort Pierce, FL 34945, USA; amir2558@ufl.edu

**Keywords:** chitosan, *Reticulitermes virginicus*, gut bacteria, 16S rRNA, Illumina amplicon sequencing, metagenomics

## Abstract

**Simple Summary:**

Termite digestion of lignocellulosic materials is aided by their gut microbial community, which includes protists, bacteria, and archaea. They play important roles in termite growth and environmental adaptation. Dark southern subterranean termites (*Reticulitermes virginicus*) are native to North America and significantly damage wooden structures. Because of their considerable economic impact, a more thorough understanding of the relationship between host termite and microbial community is needed to develop target-specific and environmentally benign wood protection systems. The overall aim of this study was to investigate the potential influence of chitosan, a biodegradable and antimicrobial compound, on termite gut bacteria. A significant effect of chitosan treatment was observed in the relative abundance of two bacteria phyla (Firmicutes and Actinobacteria). The results suggest that chitosan treatment not only affects the structure of the microbial community in the gut, but other treatments also cause shifts in termite gut communities.

**Abstract:**

A thorough understanding of microbial communities in the gut of lower termites is needed to develop target-specific and environmentally benign wood protection systems. In this study, the bacterial community from *Reticulitermes virginicus* was examined by Illumina sequencing of 16S ribosomal RNA (rRNA) spanning the V3 and V4 regions. Prior to library preparation, the termites were subjected to five treatments over an 18-day period: three groups were fed on wood treated with 0.5% chitosan, 25% acetic acid, or water, the fourth group was taken directly from the original collection log, and the fifth group was starved. Metagenomic sequences were analyzed using QIIME 2 to understand the treatments’ effects on the dynamics of the gut bacteria. Four dominant phyla were detected: Bacteroidetes (34.4% of reads), Firmicutes (20.6%), Elusimicrobia (15.7%), and Proteobacteria (12.9%). A significant effect of chitosan treatment was observed in two phyla; Firmicutes abundance was significantly lower with chitosan treatment when compared to other groups, while Actinobacteria was lower in unexposed and starved termites. The results suggest that chitosan treatment not only affects the structure of the microbial community in the gut, but other treatments such as starving also cause shifts in termite gut communities.

## 1. Introduction

Termites are social insects belonging to the order Blattodea. Termites are divided into phylogenetic lower termite and higher termite lineages. The major difference between these two termite subgroups is the unique presence of symbiotic protists in lower termite gut, which are absent in the gut of higher termites. Termites in the United States are generally categorized based on their living habitat as subterranean, drywood, or dampwood. Subterranean termites (Rhinotermitidae) are lower termites that have a significant economic impact on wood structures in the United States. Within the subterranean termites, *Reticulitermes flavipes* (Kollar), *Reticulitermes virginicus* (Banks), *Reticulitermes hesperus* (Banks), *Reticulitermes hageni* Banks, *Reticulitermes tibialis* Banks, and *Coptotermes formosanus* Shiraki cause the majority of termite damage to wooden, human-built structures [1]. Both *R. flavipes* and *R. virginicus* are commonly found in forest and residential areas in Mississippi [2]. *R. flavipes* is known as the eastern subterranean termite, and *R. virginicus* as the dark southern subterranean termite. The damage of *R. virginicus* was assumed to be insignificant until 1990 when Su and Scheffrahn [1] reported a high rate of infestation of structures in Florida. Because of the morphological similarity between *R. flavipes* and *R. virginicus*, structures infested by *R. virginicus* are believed to be identified incorrectly. Consequently, *R. virginicus* attracted less attention.

In lower termites, the prokaryotes (bacteria and archaea) and the eukaryotes (protists) in the hindgut are essential for host survival. The hindgut microbial symbionts are horizontally transferred to nestmates in the termite colony via proctodeal feeding [3]. Although 90% of hindgut volume is inhabited by protists (10^3^ to 10^5^ cells per single gut) that play an important role in the digestion of cellulose, the bacterial community is also present in high abundance (10^6^ to 10^8^ cells per single gut), and are thought to be fundamental for lignocellulose degradation [4]. Even though bacteria are not significantly involved in cellulose digestion directly, they maintain the essential chemical environment through specific processes ascribed to acetogenic bacteria, spirochetes (homoacetogenic and oxygenase activity), nitrogen-fixing bacteria, lactic acid bacteria, sulfate-reducing bacteria, and uric acid-degrading bacteria [5,6,7,8]. Symbiotic bacteria in the hindgut of lower termites have been shown to be associated with the protists, attached to the gut wall, or present as free-living cells in the hindgut lumen. In general, most bacteria are associated with the surface (ectosymbionts), cytoplasm (endosymbionts), or nucleus (endosymbionts) of protist species [3]. Since part of lumen contents are transferred among nestmates, there is a possibility of a washout of some microorganisms, which is believed to be overcome either by their fast motility or attachment to non-passing particles [3]. Nakajima et al. [9] reported differential distribution of bacterial communities between luminal content (including free bacteria or those associated with gut protists) and gut wall in *Reticulitermes speratus* (Kolbe) through culture-independent methods. Bacteria from the Actinobacteria, Firmicutes, and Bacteroidetes phyla were predominantly associated with the gut wall, while the ones belonging to the Spirochaetes and Termite Group I phyla were rare in the gut wall.

Since culturing techniques for bacterial symbionts require a strict cultivating environment for survival and reproduction, culture-independent methods make it possible to determine phylogenetic diversity of the termite gut bacteria [10]. Ohkuma and Kudo [11] studied amplified partial 16S ribosomal RNA (rRNA) genes from mixed microbial DNA of the *R. speratus* hindgut. Most bacteria in the hindgut were affiliated with five groups of bacteria, including the Cytophaga–Flexibacter–Bacteroides group, the low-G+C Gram-positive bacteria, Proteobacteria, Spirochaeta, and Termite Group I. Later on, Hongoh et al. [12] examined the phylogenetic diversity of bacteria in the *R. speratus* hindgut through sequence analysis of near full-length 16S rRNA gene clones. This study revealed many phylotypes for the first time: Spirochaetes, Firmicutes, Bacteroidetes, Actinobacteria, Proteobacteria, Planctomycetes, Verrucomicrobia, Cyanobacteria, Acidobacteria, and other rare phyla, among which the spirochetes were the most prevalent. A taxonomy comparison of wood-feeding bacteria in lower termites showed that the phylum Spirochaetes was dominant in the hindgut of *R. flavipes* and *R. speratus,* while the phylum Bacteroidetes represented the major bacteria species in *C. formosanus* [7,10].

The diversity of bacteria in termite hindguts has been attributed to a variety of factors. Ecology and genetics seem to have the major influence on the termite hindgut bacterial community, which can be highly diverse, with thousands of species detected in *R. flavipes* [13]. Although the alpha diversity of the protists and bacteria of lower termites were found to be correlated, protist diversity is more phylogenetically dependent, while the bacterial population is more influenced by ecology [14]. Diet does not have a pronounced effect on the major phyla of bacterial communities, but it does influence their composition. Huang et al. [15] reported that the microbial composition of *R. flavipes* fed on woody and grassy diets changed for each diet type. Benjamino et al. [16] showed that a change in diet mostly affected less abundant bacteria, and pointed to the findings of others that these non-core bacteria can have a crucial role in the metabolic process during unfavorable conditions.

Chitosan, an oligosaccharide known for its antimicrobial properties, is made from chitin, which is isolated mainly from the outer exoskeleton of arthropods (including crustaceans and insects), marine diatoms, algae, fungi, and yeasts [17,18]. The antifungal actions of chitosan have been shown to inhibit the growth of brown rot (*Poria placenta* and *Coniophora puteana*) and white rot fungi (*Coriolus versicolor*). The high molecular weight of chitosan was found to be efficient against wood decay fungi [19]. Moreover, the effectiveness of chitosan on inhibiting the growth of Gram-positive and Gram-negative bacteria has been reported in several studies [20,21,22]. The exact mechanism of its activity, though, is not completely understood.

Despite its significant antimicrobial properties, chitosan has low toxicity to mammalian cells and non-target organisms. Current trends in wood protection research aim to find alternative biocides that pose little threat to non-targeted species and exhibit low persistence and accumulation in the environment. For instance, the biodiversity of aquatic organisms is negatively impacted by high levels of heavy metals, such as copper, which is currently the major component of wood preservatives used in both residential and industrial applications [23]. Thus, chitosan may serve as a potentially viable alternative to copper in wood preservation [24].

In our previous study, Raji et al. [25] showed that wood treated with chitosan was toxic to both *R. virginicus* and *R. flavipes*, causing a dose-dependent effect on termite mortality. A significant disappearance of protists was observed in the hindgut of the chitosan-fed *R. virginicus* [26]. The antimicrobial effects of chitosan on the termite hindgut bacteria, though, have not yet been described. Therefore, the present study continues our evaluation of chitosan as a potential termiticide for wood treatment by examining its effects on the hindgut bacterial diversity of *R. virginicus.* Bacterial species composition and relative abundance were evaluated by sequencing the V3-V4 hyper-variable regions of the 16S rRNA gene using the Illumina MiSeq platform. These results should provide a more complete understanding of how chitosan exerts its toxicity on the termite host.

## 2. Materials and Methods

### 2.1. Termite Collection and Species Identification

Termites were collected from a single infested pine log found at the Mississippi State University Dorman Lake Test Site in Starkville, Mississippi (May 2017). The log was cut into small sections and placed into two covered metal (32-gallon) containers. The containers were brought back to the laboratory and maintained at room temperature in the dark. Workers were sampled two times, immediately after the field collection to characterize the hindgut bacterial community from the natural habitat, and again two months later to obtain individuals for the chitosan study.

Species-level identification of the termites was performed via morphological analysis using the identification guide of Messenger [27], and DNA sequence analysis of the mitochondrial AT-rich region [28]. For the latter, genomic DNA was extracted from five soldier heads using the MasterPure TM Complete DNA and RNA Purification Kit (Epicentre, Madison, WI, USA), and amplified in a polymerase chain reaction (PCR) using the primer sequences described by Foster et al. [28]. The purified PCR fragment (~400 bp) was cloned using the pGEM-T Easy Vector System II kit (Promega, Madison, WI, USA). Plasmid DNA was isolated from four recombinant clones according to the PureLink Quick Plasmid Miniprep Kit (Invitrogen, Carlsbad, CA, USA) and sent for sequencing to Eurofins Genomics (Louisville, KY, USA). The obtained DNA sequences were trimmed from the vector and aligned in a BLASTN search [29] against the National Center for Biotechnology Information (NCBI) non-redundant (nr) nucleotide database to find species matches with the greatest percent similarity.

### 2.2. Wood Treatment and Termite Feeding

Low-molecular-weight chitosan powder (50–190 kDa) was purchased from Sigma-Aldrich (St Louis, MO, USA). To prepare the 0.5% chitosan (*w*/*v*) solution, chitosan (0.5 g) was dissolved in 25% aqueous acetic acid (*v*/*v*) to achieve a final volume of 100 mL, pH 1.85.

Defect-free southern yellow pine end-matched sapwood samples measuring 25 × 25 × 6 mm (tangential × radial × longitudinal) were oven dried at 50 °C to a constant weight. A total of fifteen oven-dried wood samples were randomly chosen, and a set of five were submerged in 100 mL of different treatment solutions: 0.5% chitosan, 25% acetic acid (*v*/*v*), and distilled water. The samples were vacuum treated at 29.8 mm Hg for 3 h, after which the vacuum was broken and the samples were subsequently equilibrated in the solutions for 24 h. Samples were taken out from the solutions, gently wiped, and allowed to air dry at room temperature for several hours, and then once again, oven dried to constant mass. In addition, termite-exposed treated wood samples were similarly dried in a laboratory oven (50 °C) to constant mass, and the values were used to determine wood percent mass loss after termite exposure, as follows:

(1) $$\mathrm{Mass}\;\mathrm{loss}\;(\%)\;=\;\frac{\left(m_{\mathrm{ot}}-m_{\mathrm{ote}}\right)}{m_{0t}}\times 100$$

where m_ot_ is the oven-dried mass of chitosan treated wood samples and m_ote_ is the oven-dried mass of treated wood samples after termite exposure.

Moreover, retention of chitosan in treated wood samples was calculated using the following equation:

(2) $$\mathrm{Retention}\;(\mathrm{mg}\;g^{-1})\;=\;\frac{\left(m_{\mathrm{ot}}-m_{o}\right)}{m_{o}}\times 1000$$

where m_ot_ (g) is the oven-dried mass of chitosan-treated wood and m_o_ (g) is the oven-dried mass of samples before treatment.

A total of 20 round-bottom glass jars (8 cm wide, 10 cm tall) each containing 150 g play sand (Quikrete Premium Play Sand) and 24 mL distilled water were autoclaved for 45 min. Upon cooling, one treated wood block and 1 g of worker termites, which contained approximately 300 workers and three soldiers, were placed on top of the play sand of 15 jars. In five jars, which were used for starvation effects, only 1 g of termites was placed. Thus, the study included four treatments with five replicates: 25% acetic acid-treated wood-exposed termites (ACE), water-treated wood-exposed termites (WE), 0.5% chitosan-treated wood-exposed termites (CTE), and starved termites (STV). After 18 days, all surviving workers were collected, cleaned, and stored at −80 °C until DNA isolation could be performed. As noted above, unexposed termites (UNX) were immediately placed into the freezer (−80 °C) on the day of field collection, and they were used in the analysis as the fifth treatment.

### 2.3. Termite Dissection and DNA Isolation

Guts were removed from the 60 termites per replicate jar by pulling the tip of the abdomen from the thorax with forceps to release the digestive tract from the termite exoskeleton. The guts were washed in a droplet of PBS buffer solution (130 mM NaCl, 10 mM sodium phosphate buffer, pH 7.2). Five guts were placed in one of 12 microcentrifuge tubes per replicate jar. The MasterPure^TM^ Complete DNA and RNA Purification Kit protocol was followed for genomic DNA extraction. From the 12 microcentrifuge tubes, four microcentrifuge tubes of the extracted DNA were randomly pooled into one DNA subsample, so a total of three subsamples were obtained per replicate jar, making a total of 15 DNA subsamples per treatment, and a total of 75 DNA subsamples for the study (i.e., five treatment groups).

RNA was removed from the extracted DNA by treatment with RNase A at 37 °C for 15 min, and the RNase A-treated samples were purified using a DNeasy Blood and Tissue Kit (Qiagen, Germantown, MD, USA). Concentration of the genomic DNA subsamples were assessed by a NanoDrop^TM^ spectrophotometer. The quality of the genomic DNA samples was examined by agarose gel electrophoresis.

### 2.4. 16S Amplification and Library Preparation

To prepare a bacterial metagenomics library, the 16S Metagenomic Sequencing Library Preparation Guide (Illumina Part #15,044,223 Rev. B) was followed. In summary, 2.5 µL of the genomic DNA diluted to 10 ng/µL in 10 mM Tris pH 8.5 were amplified using forward and reverse gene-specific primer sequences developed by Klindworth et al. [30] for the V3 and V4 hyper-variable regions of the 16S rRNA gene. Amplification was conducted using a thermal cycler program of: 95 °C for 3 min, followed by 25 cycles of 95 °C (30 s), 55 °C (30 s), and 72 °C (30 s), with a final extension step of 72 °C for 5 min. The amplicon PCR products were separated by gel electrophoresis on a 1.5% agarose gel in 1× Tris–acetate–EDTA (TAE) buffer to confirm amplification specificity and size (~550 bp). The amplicon PCR products were purified with AMPure XP (Beckman Coulter, Indianapolis, IN, USA) magnetic beads, and the purified products were indexed with a unique combination of Illumina Nextera XT Index Primer 1 and 2 according to the library preparation guide. Index PCR products were also purified by AMPure beads.

Libraries were quantified by a Qubit dsDNA HS assay (Life Technologies: Molecular Probes32851 Rev. B) using a Qubit 1.0 fluorometer (Thermo Fisher Scientific, Waltham, MA, USA). A Bioanalyzer DNA 1000 chip (Agilent Technologies, Santa Clara, CA, USA) was used to verify the expected size of approximately 630 bp. Concentrations of each library were calculated and then diluted to 20 nM using 10 mM Tris-HCl (pH 8.5) containing 0.1% Tween 20. Subsequently, 2 μL of each 20 nM library from all 75 samples were pooled into one tube and submitted for sequencing on a MiSeq system using the 600-cycle MiSeq Reagent Kit v3 (Illumina, San Diego, CA, USA). Bioanalyzer analysis and MiSeq sequencing were performed by personnel at the Institute for Genomics, Biocomputing and Biotechnology (IGBB) at Mississippi State University.

### 2.5. Sequencing, Data Processing, and Analysis

The MiSeq output contained demultiplexed FASTQ sequence files corresponding to the individual sample libraries in the submitted poolplex tube. MiSeq sequencing resulted in 19 million total raw reads obtained from 75 samples. Raw read sequence data were archived at NCBI as Sequence Read Archive (SRA) accession PRJNA648009. After demultiplexing, 14,625,126 reads were obtained (77% of total reads). Data analysis was performed using the open source software Quantitative Insights Into Microbial Ecology (QIIME 2 version 2018.8) [31]. The DADA2 plugin [32] was used to remove the PhiX (adapter-ligated library) control reads and chimeric sequences. The reads displaying high-quality scores (≥20) were chosen and all forward and reverse sequences were trimmed from position 14 to 252 bp. After the filter and trim steps, 11,320,858 (60% of total) reads remained. In addition, the DADA2 plugin was used to cluster unique sequence variants, which are similar to operational taxonomic units (OTUs) and provide counts (frequencies) of each unique sequence in each sample.

For alpha diversity estimation, sample richness and Shannon’s diversity index were calculated. Species richness was measured as the number of different species (observed OTUs) present in a sample. The sequence counts were subsampled at a sequencing depth of 200,000 reads to drop off counts with a lower sampling depth from the diversity analysis, which resulted in retaining 44.17% of sequences in 100% of total samples. Species diversity was calculated for each treatment community using Shannon’s diversity index to discern both species richness and relative abundance of different species (species evenness) [33].

Pielou’s evenness was used to determine species evenness, which explains how evenly the relative abundance of species is distributed in a sample [34]. Evenness is restricted between 0 and 1, in which the higher value suggests the maximum evenness and vice versa.

For beta diversity, a principal coordinate analysis (PCoA) plot of sequence counts was constructed using Bray–Curtis dissimilarity [35] to visualize differences in the microbial abundance from different treatments. Permutational multivariate analysis of variance (PERMANOVA) statistical analysis was performed using the Bray–Curtis distance metric in QIIME 2 and then followed by pairwise comparison analysis between treatment groups. This analysis performed over 999 permutations and provided a pseudo-F, *p*-value (*p*), and group significance plots. Sequences were then taxonomically classified using a naive Bayes classifier pre-trained on the Dictyopteran gut microbiota reference database (DictDb) (v. 3.0, 2015) [36]. A heatmap was constructed using an average clustering method with a Euclidean metric for hierarchical clustering of sequence data to show the abundances of bacteria among samples in different treatments at the phylum level.

To assess bacterial relative abundance at the phylum level and genus level, rare sequences were first removed. Rare sequence counts for both taxonomic levels were defined as sequences seen in either fewer than three biological replicates (total five replicates per treatment) or as a total count of fewer than 10 sequences in all replicates. The relative abundance was calculated by dividing individual sequence counts for a phylum or genus by the total sequence counts in a sample (without the rare sequences) and then multiplying by 650,000 (which was obtained as the highest total sequence count among all samples) [37,38]. Statistical Analysis Software version 9.4 (SAS Institute Cary, NC, USA) was used to perform one-way analysis of variance (ANOVA) to determine differences for each classification level across the treatment groups and to calculate mass loss of treated wood samples exposed to termites. A Shapiro–Wilk test was used for a data normality check, and homogeneity of variance was assessed by Levene’s test. If the assumption of homogeneity of variance was met, which means the *p*-value was greater than the 0.05 significance level, ANOVA was performed with Tukey’s post hoc test. The false discovery rate (FDR < 0.05) was found to correct for multiple ANOVA testing.

## 3. Results

### 3.1. Termite Species Identification and Feeding Bioassay

Alignment of the colony termites’ sequences against the NCBI nr nucleotide database through BLAST confirmed termite species identification as *R. virginicus* (100% coverage and 99% identity). Phenotypically, the size of the rectangular-shaped head capsule and the inward curvature of the right mandible in soldiers also supported termite identification as *R. virginicus*.

A visual estimation of termite death displayed less than 50% mortality in termites subjected to wood samples with ≤ 29 mg g^−1^ chitosan retention (0.5% chitosan, CTE), while approximately 25% mortality was observed in termites exposed to controls (water and 25% acetic acid, WE and ACE). More than 90% mortality was estimated for the starved termite group (no wood block, STV). Even though the termite mortality for the CTE group was 2× higher than for the control groups, the average percent mass loss of chitosan-treated wood samples after exposure to termites was not significantly different from the controls (Table 1).

### 3.2. Treatment Effects on Hindgut Bacterial Diversity

Shannon’s diversity index showed that there was a significant difference between species diversity of UNX and other treatments. While the mean UNX Shannon’s index of 8.16 was significantly higher, the mean Shannon’s index of other treatments ranged from 6.43–6.76 with no significant differences among treatments (Table 2). Moreover, the highly diverse bacterial community in UNX samples was corroborated by the observed significantly higher mean species richness of 1823 OTUs and Pielou’s evenness of 0.75. Lower Shannon’s indices for the other treatments indicated a loss of species diversity and richness due to the changed termite environment and the effectiveness of termite diet.

**Figure 1:**
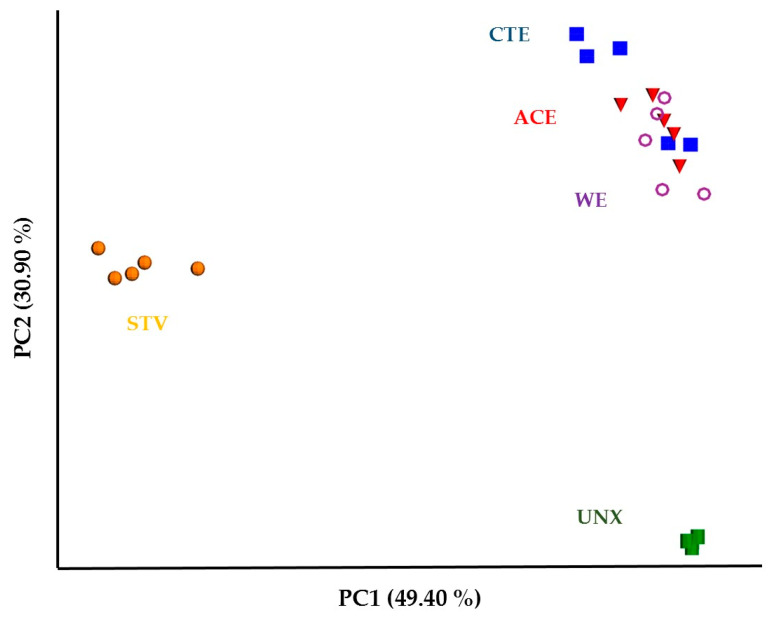
Principle coordinate analysis (PCoA) of a Bray–Curtis dissimilarity matrix from the hindgut bacteria detected in *R. virginicus* exposed to five groups. Each group is represented by a unique color and shape.

Beta diversity showed more clear differences between the treatments, as a PCoA plot of bacterial sequence counts from five treatments revealed three cluster groups with different spatial ordinations. STV and UNX formed two distinct clusters (Figure 1). Although bacterial communities (observed bacteria in the termite gut) in CTE, ACE, and WE were allocated within the same cluster, some CTE replicate samples showed a slight divergence of bacterial sequence counts compared to others. PERMANOVA indicated that there was a significant effect of treatment on bacterial diversity in the termite gut (*p* = 0.001; Table 3). Pairwise pseudo-F test statistic comparisons revealed that bacteria compositions were significantly different among the samples except in the case of CTE and ACE, as shown in Table 3, which could have resulted from the presence of acetic acid in these wood samples, or the low concentration of chitosan used in the study.

Among 5144 unique OTUs, 3004 OTUs were identified as rare OTUs, and included 11 OTUs that appeared in fewer than three samples but had a high frequency of over 500 observed sequences. The BLAST of these 11 rare OTUs against the NCBI nr nucleotide database assigned four OTUs to Proteobacteria, three OTUs to the phylum Firmicutes, and the remaining OTUs to Actinobacteria, Spirochaetes, Bacteroidetes, and Elusimicrobia, with a range of 94 to 100% identity. The 11 rare OTUs had perfect BLAST matches with organisms that were isolated from the termite gut, environmental sample, sewage sludge, and soil. Therefore, there is a possibility that these 11 OTUs are not rare but have incredibly uneven distributions among samples.

A total of 28 phyla, 50 classes, 101 orders, 190 families, and 409 genera were classified against the DictDb v. 3.0 database. Among 28 phyla, rare phyla were assigned to Fusobacteria, Fibrobacteres, Chloroflexi, Candidate phylum BRC1, and Armatimonadetes. The proportional presence of major phyla in all treatments as calculated upon the removal of rare phyla was found in the following order: Bacteroidetes (34.4% of reads), Firmicutes (20.6%), Elusimicrobia (15.7%), Proteobacteria (12.9%), and Spirochaetes (8.2%).

**Figure 2:**
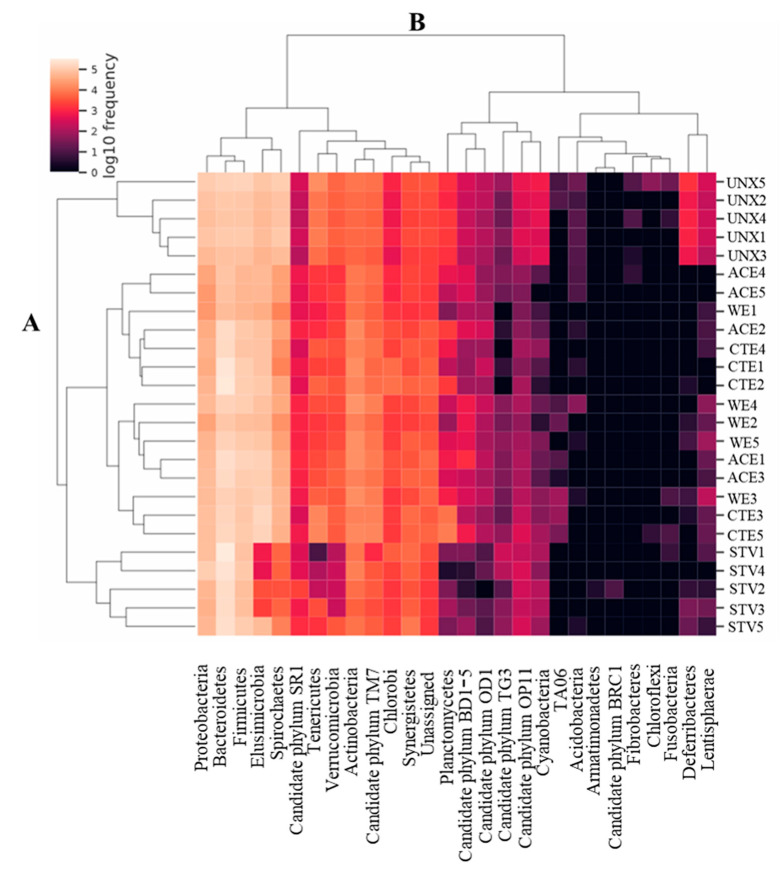
Two-way cluster analysis showing abundance of bacterial phyla per treatment in the hindgut of *R. virginicus*. Dendrogram A, sample clustering; dendrogram B, phylum clustering; TA06, candidate phylum TA06. The color bar in the top left indicates abundance; light color, high; dark color, low.

Two-way cluster analysis based on Euclidean distance calculated from the normalized log_10_ sequence read counts was further used to examine the relationship among the samples and phyla (Figure 2). Dendrogram A showed that the samples formed two major clusters, one containing all UNX samples, while the other comprised all remaining samples. The latter included two major sub-clusters, where STV samples formed a separate branch from WE, ACE, and CTE. Dendrogram B for the phyla analysis also revealed two major clusters, and each one of them contained two main sub-clusters (Figure 2). One cluster contained 13 bacterial phyla of higher abundance than the second cluster containing 15 phyla. The predominant bacterial phyla in the high-abundance cluster were Bacteroidetes, Firmicutes, Proteobacteria, Elusimicrobia, and Spirochaetes.

**Figure 3:**
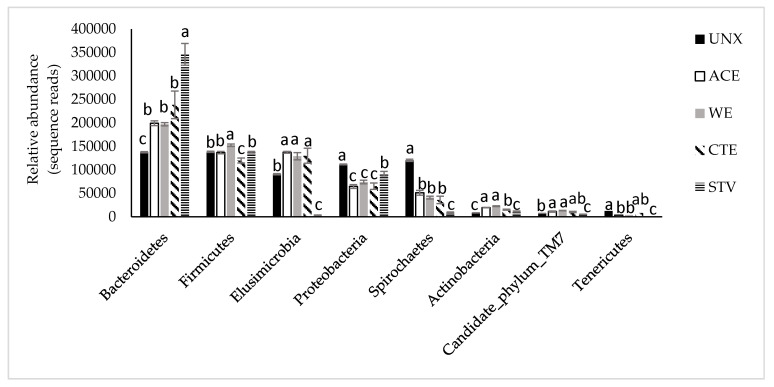
Effect of treatment on relative abundance of bacterial phyla. Bars represent means ± SE. Different letters above error bars within each phylum represent significant treatment differences, which were obtained by ANOVA followed by a multiple comparisons test corrected for false detection rate (FDR).

The abundances of the eight phyla with the highest read counts were examined in detail among treatments and are shown in Figure 3. Bacteroidetes was the most abundant phylum in all treatments and seemed to especially proliferate in STV samples. In contrast, Bacteroidetes were present in the lowest amounts in UNX samples. UNX had significantly higher relative amounts of Proteobacteria and Spirochaetes when compared to the other four treatments, and higher relative amounts of Tenericutes than ACE, WE, and STV samples (Figure 3). This would indicate that the change in diet had the most profound negative effect on these three phyla. Relative amounts of Elusimicrobia and Actinobacteria were positively affected by the treatments, as they were significantly higher in ACE, WE, and CTE samples. STV samples exhibited the lowest amounts of five phyla: significantly lower amounts of Elusimicrobia, Spirochaetes, Candidate phylum TM7, and Tenericutes compared to the other four phyla and shared the lowest amounts of Actinobacteria with UNX samples. UNX and STV also had a similar relative abundance of Firmicutes. Firmicutes seemed to be the most affected by chitosan treatment, showing the lowest relative amounts in CTE samples.

Therefore, CTE, ACE, and WE groups had similar relative amounts of most of the presented phyla, except Actinobacteria, which was significantly less abundant in CTE samples. Candidate phylum OP11 and Verrucomicrobia also showed similar amounts in these three groups, but are not presented in the chart.

At the genus level, all the rare genera were removed and subsequently the counts of sequence reads from 254 bacteria were statistically evaluated. The influence of chitosan treatment distinctly affected the relative abundance of only 36 genus-level bacteria (Table 4), with the majority of those affected belonging to Firmicutes (72.2%), Proteobacteria (8.3%), Bacteroidetes (5.5%), Actinobacteria (5.6%), Candidate phylum BD1-5 (2.8%), Candidate phylum TM7 (2.8%), and Tenericutes (2.8%). Among these 36 genus-level identified bacteria, 16 (order number 1–16, Table 4) showed the lowest relative amount in CTE samples, and seven (17–23, Table 4) showed the lowest amount in CTE and STV samples. An additional eight (24–31, Table 4) of the genus-level bacteria showed the lowest relative amounts in STV samples, with chitosan being the second major cause for a significant reduction in their relative amounts.

Conversely, *Lactococcus* 1 (non-opportunistic pathogen) had the highest relative amount in the CTE samples, and *Corynebacterium* 5 (occasionally an opportunistic pathogen), being the most abundant in STV samples, showed the second highest amount in CTE samples (32–33, Table 4). Besides these, Termite cluster 3 from family Porphyromonadaceae 2 (Bacteroidetes) and Termite cockroach cluster 1 from Family XIII Incertae Sedis (Firmicutes) showed an increase in relative abundance in CTE samples when compared to UNX and STV samples. Moreover, Termite cluster III (Proteobacteria) displayed a higher abundance in CTE and STV when compared to ACE and WE.

## 4. Discussion

The 18 days of exposure of *R. virginicus* to the low level of chitosan retention (29 mg g^−1^; 0.5% chitosan solution; CTE) resulted in 50% termite mortality and 23% wood mass loss. Raji et al. [25] obtained 100% mortality and 5% mass loss in a study of *R. virginicus* exposed to the chitosan treatment with a lower retention (11 mg g^−1^) for a longer period of time (28 days). Comparing termite mortality in a 0.5% chitosan treatment with controls (water and 25% acetic acid) between the two studies indicated that the study of Raji et al. [25] also had 2.2× higher mortality for the termites exposed to the same type of controls for 10 days longer than our samples. Since the retention in the chitosan-treated wood was higher in this study than in the study of Raji et al. [25], the lower mortality and higher mass loss of this study could be attributed to differences in colony vigor, time and location of colony collection, or the number of termites used in the experiments. No difference in average mass loss observed between chitosan treatment and controls could be assigned to the low concentration of chitosan treatment, which did not prevent termites from consuming wood, or fewer numbers of termites in the chitosan jar eating the same amount of food as in the control containing 50% more termites due to less mortality.

Chitosan, a recognized antimicrobial polymer, can be formed by chitin deacetylase enzyme (CDA), which is a hydrolytic enzyme, in the termite gut. It may also influence the binding of proteins to chitin, which is the main component of the peritrophic matrix (PM), and alter the permeability, elasticity, and strength of the PM [39]. The PM plays an important role in food digestion and protection of the gut epithelium against pathogens and toxins. Sandoval-Mojica and Scharf [40] identified and characterized gut genes in *R. flavipes* that produce the main components of the PM. To encode CDA in *R. flavipes*, three genes, *RfCDA1*, *RfCDA2*, and *RfCDA3*, were identified [40]. Two of three deacetylases expressed by the hindgut bacterial symbionts (*Rf*CDA1 and *Rf*CDA3) are not associated with termites, while the other one showed a high percentage of similarity with an insect CDA. Bacterial symbiont deacetylase may lead to the production of chitosan to provide termites with a nitrogen food source or defense mechanism to control pathogenic bacteria. Sandoval-Mojica and Scharf [40] suggested the potential of *RfCDA2* as termiticide target in future. Any change in the components of the PM may affect cuticle structure or formation in termites and subsequently lead to termite mortality. One possible reason for the high mortality of termites exposed to high concentrations of chitosan could be associated with a change in a ratio of chitosan to chitin (which may directly or indirectly affect the binding of proteins to chitin) in different parts of gut. When feeding *R. virginicus* on the wood treated with high concentrations of chitosan (1% and 2%), eight out of ten protist species found in the hindgut were diminished [26]. These protists were previously reported to be associated with different bacterial ecto- and/or endosymbionts [41,42,43]. The only two protists that remained detectable, *Monocercomonas* sp. and *Trichomitus trypanoides* (Dubosq and Grassé), are believed not to have symbiotic associations with bacteria [44,45]. It is possible that the effect of chitosan on the protist or bacteria community results from instability of the microbial community in the hindgut. This study also reported that all ten protists were present in the hindgut of termites fed on wood treated with a low concentration of chitosan (0.5% chitosan) [26]. Thus, using sublethal chitosan (0.5%) in *R. virginicus* was an adequate concentration to determine bacterial diversity, even though protists were not clearly affected by the 0.5% chitosan treatment. In the current study, termites tested soon after collection from the forest showed the most abundant phyla to be Bacteroidetes (6.1%), Firmicutes (6.1%), Spirochaetes (5.3%), Proteobacteria (4.9%), and Elusimicrobia (4.0%). Similarly, *R. virginicus* fed on a natural wood diet, collected from Florida, also showed the most abundant bacteria to be Spirochaetes (18.9%), Elusimicrobia (10.2%), Proteobacteria (5.9%), Firmicutes (4.7%), and Bacteroidetes (4.5%) [46,47], although the relative amounts of each phylum differed between the two studies. The differences in phyla abundance could be attributed to different termite colonies, physiology, maintenance, diet, and local environment, which are important factors in shaping the gut microbiota compositions.

Benjamino et al. [16] reported a shift in core bacteria resulting from a loss of symbiotic protists only in starved *R. flavipes* colonies. Feeding *R. flavipes* on a new diet caused shifts in the microbial community after 7 days. Our study also revealed a significant effect of diet on the bacterial diversity in the gut of *R. virginicus*. Although we did not perform a temporal study, the differences in bacterial diversity were observed after 18 days of exposure to new diets. Higher microbial diversity is expected in UNX since termites in their natural habitat forage different food sources and acquire new microorganisms as well as micronutrients from soil [48,49].

Tang et al. [50] found that when *R. flavipes* was fed chitosan-treated wood, disease-causing pathogens became established in the gut, thereby suggesting that antimicrobials like chitosan cause host mortality by inducing dysbiosis or long-term microbial imbalance. Using an indicator species analysis, they found that nine species were specific to the 2% chitosan treatment. Among these nine indicators, three species, *Mycobacterium abscessus* and *Mycobacterium franklinii* (Actinobacteria) and *Sphingobacterium multivorum* (Bacteroidetes), were found to be opportunistic pathogens based on their literature review. Feeding subterranean termites on different antibiotic treatments has been investigated by several studies [47,51]. Four antibiotic treatments (ampicillin, kanamycin, metronidazole, and tetracycline) induce dysbiosis in *R. flavipes* and cause a significant decrease in symbiont abundance and diversity after 7 days of antimicrobial feeding [51]. In that study, the maximum biodiversity and evenness were shown in the bacterial community of termites feeding on non-treated wood samples, which is in agreement with our findings in the *R. virginicus* UNX group.

Peterson et al. [51] indicated that kanamycin reduced the bacterial diversity and evenness, compared with the other three antibiotic treatments. Although the result of our study for the CTE group showed the least diversity and evenness values, similar to the kanamycin treatment, there was no statistically significant difference between CTE and the two other groups (WA and ACE). Interestingly, kanamycin and metronidazole both displayed an increase in the relative abundance of Bacteroidetes and a decrease in Firmicutes compared with non-treated samples and the two other antibiotic treatments (ampicillin and tetracycline). The latter finding is comparable with the results found in our study for the CTE group, even though the termite species differed. However, the STV group had the highest relative abundance in Bacteroidetes compared to other treatments, while the effect of antibiotic treatments in the Peterson et al. [51] study was not tested on termites without a food source (starvation). In the current study, the abundance of Firmicutes in the STV group did not show any significant difference from UNX. Therefore, we can assume that the effect of kanamycin and chitosan in Bacteroidetes may induce a higher degree of dysbiosis in the termite gut. Conversely, feeding a ciprofloxacin antibiotic to *Reticulitermes grassei* over a seven-day period resulted in smaller proportions of Bacteroidetes but the change in the microbial community in that study was not significant, leading to the establishment of opportunistic pathogens [47]. The conflict between these studies cannot be ascribed to the difference among *Reticulitermes* species because of similar values of the community evenness described in Berlanga et al. [47]. It may be explained by differences in antimicrobial sources and various enzymatic biodegradation pathways, for instance, as the latter paper stated that ciprofloxacin appeared to influence xylene degradation, nitrogen fixation, and xenobiotic biodegradation.

A study of hindgut bacteria in *R. flavipes* as affected by chitosan-treated wood also showed predominant phyla within chitosan-treated and water-treated (control) groups to be Bacteroidetes, Firmicutes, Elusimicrobia, and Proteobacteria [52]. The last two phyla showed a significant change—Proteobacteria saw a decrease and Elusimicrobia an increase in *R. flavipes* fed on chitosan-treated wood. These results are different than the observation of our study on *R. virginicus*, which indicated no significant difference between CTE and WE samples, and an opposite chitosan effect when comparing CTE to UNX samples—an increase in Proteobacteria and a decrease in Elusimicrobia. Raji et al. [52] showed no differences between WE and CTE samples in *R. flavipes* for the Bacteroidetes and Firmicutes phyla, similarly to our study, but we observed a significant loss of Firmicutes in CTE samples compared to both UNX and WE in the case of *R. virginicus*. The differences between the studies could be explained by differences in termite and bacterial species, the design of the experiment, and data analysis.

The relative abundance of Proteobacteria and Bacteroidetes increased and the amount of Spirochaetes decreased in *Tsaitermes ampliceps* (known as *Reticulitermes ampliceps* Wang and Li) fed on a lignin-poor diet for two weeks when compared to termites fed on a lignin-rich diet [53]. We also saw an increase in Bacteroidetes in STV compared to all other samples, and in Proteobacteria when STV was compared to ACE, CTE, and WE samples. Spirochaetes were also significantly lower in STV samples in comparison to all other samples. These results confirm the findings of Tokuda et al. [54] that Spirochaetes have a very important role in the lignocellulose degradation of wood-feeding termites, while Bacteroidetes and Firmicutes bacteria appear to be very important in producing mycolytic enzymes in the hindgut of fungus-growing termites [55].

Besides the expected shift in bacteria driven by enzymatic compatibility with the substrate, the presence of chitosan was expected to change bacterial populations due to the sensitivity of bacteria to the antimicrobial properties of chitosan. No et al. [23] showed the effectiveness of 0.1% chitosan to be higher against Gram-positive than Gram-negative bacteria. On the other hand, 0.02% low-molecular-weight chitosan inhibited the growth of both Gram-negative bacteria (*Escherichia coli* and *Pseudomonas aeruginosa*) and Gram-positive bacteria (*Bacillus subtilis* and *Staphylococcus aureus*) [20,56]. In our study, chitosan seemed to be effective against both types of bacteria, and among the 36 distinctly chitosan-affected genus-level bacteria, the majority were Firmicutes, which are mainly Gram positive, but identified among them were also Gram-negative Bacteroidetes, Proteobacteria, and Tenericutes.

## 5. Conclusions

The primary goal of this study is to evaluate chitosan as a future environmentally friendly pest control agent. The compositions of the bacterial communities from *R. virginicus* exposed to five diets, among them chitosan-treated wood, were characterized for the first time. Illumina MiSeq sequencing of 16S rRNA V3-V4 amplicons generated approximately 11.3 million reads. Reduction in the diversity and richness of the hindgut bacterial composition in *R. virginicus* confirmed the effect of the four treatment groups compared to naive termites. A significant effect of chitosan was observed on the abundance of 36 genus-level bacteria, the majority of them belonging to Firmicutes. Using a specific Dictyopteran sequence database (DictDb database) did not help in the identification of prokaryotes at the species level. Treatment affected the overall composition of the bacteria microbiota and the relative abundance of bacteria in the gut, detectable at the phylum and genus levels. Although chitosan is reported to be a broad-spectrum antimicrobial compound against bacteria, fungi, yeasts, and insects, which affects both Gram-positive and Gram-negative bacteria, it is shown in this study to have limited effect on specific groups of bacteria or protists in termites. The increase in opportunistic pathogens like the phylum Bacteroidetes may lead to fluctuations of other phyla in the gut, ultimately leading to termite weakness and inability to survive. The exact mechanism of the effect of chitosan on hosts and their symbionts is not fully determined and requires further studies.

## Figures and Tables

**Table 1:** Effect of treatment on wood mass loss after 18 days of exposure to *Reticulitermes virginicus*.

Treatment Solution	Percent Mass Loss * $\bar{x}$ ± SE
WE	29 ± 1.77 a
ACE	26 ± 1.53 a
CTE	23 ± 1.90 a

* Means (± SE) followed by the same lowercase letters within a column are not significantly different; WE, water-treated wood-exposed termites; ACE, 25% acetic acid-treated wood-exposed termites; CTE, 0.5% chitosan-treated wood-exposed termites.

**Table 2:** Effect of treatment on alpha diversity and evenness of hindgut bacterial communities *.

Treatment	Shannon’s Index $\bar{x}$ ± SE	Observed OTUs $\bar{x}$ ± SE	Pielou’s Evenness $\bar{x}$ ± SE
UNX	8.16 ± 0.02 a	1823 ± 63 a	0.75 ± 0.00 a
WE	6.76 ± 0.08 b	928 ± 82 b	0.69 ± 0.01 b
ACE	6.67 ± 0.05 b	900 ± 51 b	0.68 ± 0.01 b
CTE	6.43 ± 0.12 b	889 ± 61 b	0.66 ± 0.01 b
STV	6.45 ± 0.15 b	798 ± 41 b	0.67 ± 0.01 b

* Means (± SE) followed by different lowercase letters are significantly different for within column comparisons; OTUs, operational taxonomic units; UNX, unexposed termites; STV, starved termites.

**Table 3:** Permutational multivariate analysis of variance (PERMANOVA) analysis results for the significance of microbial community differences using the Bray–Curtis dissimilarity metric followed by pairwise treatment comparisons between sample groups.

Method Name	PERMANOVA
Test statistic name	pseudo-F
Sample size	25
Number of groups	5
Test statistic	24.1616
*p*-value	0.001
Number of permutations	999
Pairwise treatment groups *	UNX a WE b ACE c CTE c STV d

* Treatment groups with same lowercase letter are not significantly different.

**Table 4:** Taxonomic classification of identified genus-level bacteria and statistical differences in their relative amounts as affected by diet *.

Order No.	Phylum; Class; Order; Family; Genus
**Bacteria found in the lowest amount in CTE samples**	
1	Bacteroidetes; Bacteroidia; Bacteroidales; Porphyromonadaceae_2; Unclassified
2	Candidate phylum TM7; Termite cockroach cluster; Unclassified
3	Firmicutes; Clostridia 1; Clostridiales; ubacteriaceae 1; *Anaerofustis*
4	Firmicutes; Clostridia 1; Clostridiales; Family XIII Incertae Sedis; *Eubacterium* 3
5	Firmicutes; Clostridia 1; Clostridiales; Lachnospiraceae; *Candidatus* Arthromitus
6	Firmicutes; Clostridia 1; Clostridiales; Ruminococcaceae; Unclassified
7	Firmicutes; Clostridia 1; Clostridiales; Ruminococcaceae; *Anaerotruncus*
8	Firmicutes; Clostridia 1; Clostridiales; Ruminococcaceae; Gut cluster 5
9	Firmicutes; Clostridia 1; Clostridiales; Ruminococcaceae; Gut cluster 7
10	Firmicutes; Clostridia 1; Clostridiales; Ruminococcaceae; Gut cluster 8
11	Firmicutes; Clostridia 1; Clostridiales; Ruminococcaceae; Gut cluster 9
12	Firmicutes; Clostridia 1; Clostridiales; Ruminococcaceae; Insect cluster
13	Firmicutes; Clostridia 1; Clostridiales; Ruminococcaceae; Incertae Sedis 1
14	Firmicutes; Clostridia 1; Clostridiales; Ruminococcaceae; Uncultured 10
15	Firmicutes; Clostridia 1; Clostridiales; Ruminococcaceae; Uncultured 12
16	Firmicutes; Clostridia 2; Clostridiales 1; Peptococcaceae 1; Unclassified
**Bacteria found in the lowest amount in CTE and STV samples**	
17	Actinobacteria; Actinobacteria; Actinomycetales; Termite cluster 1; Subcluster b
18	Firmicutes; Clostridia 1; Clostridiales; Ruminococcaceae; Gut cluster 4
19	Firmicutes; Clostridia 1; Clostridiales; Ruminococcaceae; Gut cluster 6
20	Firmicutes; Clostridia 1; Clostridiales; Ruminococcaceae; Termite cockroach cluster
21	Firmicutes; Clostridia 1; Clostridiales; Ruminococcaceae; Uncultured 29
22	Firmicutes; Clostridia 2; Clostridiales 1; Peptococcaceae 2; Uncultured gut Group A
23	Tenericutes; Mollicutes; RF9; Unclassified
**Bacteria found in the lowest amount in STV samples, followed by the second lowest amounts in CTE samples**	
24	Candidate phylum BD1-5;Unclassified
25	Firmicutes; Clostridia 1; Clostridiales; Family XIII Incertae Sedis; Unclassified
26	Firmicutes; Clostridia 1; Clostridiales; Lachnospiraceae; *Catabacter*
27	Firmicutes; Clostridia 1; Clostridiales; Ruminococcaceae; *Papillibacter*
28	Firmicutes; Clostridia 1; Clostridiales; Ruminococcaceae; Termite group aaa
29	Firmicutes; Clostridia 2; Clostridiales 1; Peptococcaceae 2; *Desulfosporosinus*
30	Proteobacteria; Deltaproteobacteria; Desulfovibrionales; Desulfovibrionaceae; Gut cluster 1
31	Proteobacteria; Unclassified
**Bacteria found in the highest amount in STV samples, followed by the second highest amounts in CTE samples**	
32	Firmicutes; Bacilli; Lactobacillales; Streptococcaceae; *Lactococcus* 1
33	Actinobacteria; Actinobacteria; Corynebacteriales; Corynebacteriaceae; *Corynebacterium* 5
**Increase in relative abundance in CTE when compared to UNX and STV samples**	
34	Bacteroidetes; Bacteroidia; Bacteroidales; Porphyromonadaceae 2; Termite cluster 3
35	Firmicutes; Clostridia 1; Clostridiales; Family XIII Incertae Sedis; Termite cockroach cluster 1
**Higher abundance in CTE and STV when compared to ACE and WE**	
36	Proteobacteria; Deltaproteobacteria; Rs-K70; Termite cluster III

* For multiple hypothesis testing, FDR was tested.

## References

[1] Su N.Y., Scheffrahn R.H. (1990). Economically important termites in the United States and their control. Sociobiology.

[2] Wang C., Powell J. (2001). Survey of termites in the Delta experimental forest of Mississippi. Fla. Entomol..

[3] Brune A., Dietrich C. (2015). The gut microbiota of termites: Digesting the diversity in the light of ecology and evolution. Annu. Rev. Microbiol..

[4] Hongoh Y. (2010). Diversity and genomes of uncultured microbial symbionts in the termite gut. Biosci. Biotechnol. Biochem..

[5] Odelson D.A., Breznak J.A. (1983). Volatile fatty acid production by the hindgut microbiota of xylophagous termites. Appl. Environ. Microbiol..

[6] Potrikus C.J., Breznak J.A. (1977). Nitrogen-fixing *Enterobacter agglomerans* isolated from guts of wood-eating termites. Appl. Environ. Microbiol..

[7] Brune A. (1998). Termite guts: The world’s smallest bioreactors. Trends Biotechnol..

[8] Brune A. (2014). Symbiotic digestion of lignocellulose in termite guts. Nat. Rev. Microbiol..

[9] Nakajima H., Hongoh Y., Usami R., Kudo T., Ohkuma M. (2005). Spatial distribution of bacterial phylotypes in the gut of the termite *Reticulitermes speratus* and the bacterial community colonizing the gut epithelium. FEMS Microbiol. Ecol..

[10] Fisher M., Miller D., Brewster C., Husseneder C., Dickerman A. (2007). Diversity of gut bacteria of *Reticulitermes flavipes* as examined by 16S rRNA gene sequencing and amplified rDNA restriction analysis. Curr. Microbiol..

[11] Ohkuma M., Kudo T. (1996). Phylogenetic diversity of the intestinal bacterial community in the termite *Reticulitermes speratus*. Appl. Environ. Microbiol..

[12] Hongoh Y., Ohkuma M., Kudo T. (2003). Molecular analysis of bacterial microbiota in the gut of the termite *Reticulitermes speratus* (Isoptera; Rhinotermitidae). FEMS Microbiol. Ecol..

[13] Boucias D.G., Cai Y., Sun Y., Lietze V.U., Sen R., Raychoudhury R., Scharf M.E. (2013). The hindgut lumen prokaryotic microbiota of the termite *Reticulitermes flavipes* and its responses to dietary lignocellulose composition. Mol. Ecol..

[14] Waidele L., Korb J., Voolstra C.R., Künzel S., Dedeine F., Staubach F. (2017). Differential ecological specificity of protist and bacterial microbiomes across a set of termite species. Front. Microbiol..

[15] Huang X.F., Bakker M.G., Judd T.M., Reardon K.F., Vivanco J.M. (2013). Variations in diversity and richness of gut bacterial communities of termites (*Reticulitermes flavipes*) fed with grassy and woody plant substrates. Microb. Ecol..

[16] Benjamino J., Lincoln S., Srivastava R., Graf J. (2018). Low-abundant bacteria drive compositional changes in the gut microbiota after dietary alteration. Microbiome.

[17] Tharanathan R.N., Kittur F.S. (2003). Chitin—the undisputed biomolecule of great potential. Crit. Rev. Food Sci. Nutr..

[18] Raafat D., Sahl H.G. (2009). Chitosan and its antimicrobial potential-a critical literature survey. Microb. Biotechnol..

[19] Eikenes M., Alfredsen G., Christensen B., Militz H., Solheim H. (2005). Comparison of chitosan with different molecular weights as possible wood preservative. J. Wood Sci..

[20] Takahashi T., Imai M., Suzuki I., Sawai J. (2008). Growth inhibitory effect on bacteria of chitosan membranes regulated with deacetylation degree. Biochem. Eng. J..

[21] Goy R.C., Britto D.D., Assis O.B.G. (2009). A review of the antimicrobial activity of chitosan. Polimeros.

[22] No H.K., Park N.Y., Lee S.H., Meyers S.P. (2002). Antibacterial activity of chitosans and chitosan oligomers with different molecular weights. Int. J. Food Microbiol..

[23] Tarras-Wahlberg N.H., Flachier A., Lane S.N., Sangfors O. (2001). Environmental impacts and metal exposure of aquatic ecosystems in rivers contaminated by small scale gold mining: The Puyango River basin, southern Ecuador. Sci. Total Environ..

[24] Liibert L., Treu A., Meier P. (2011). The fixation of new alternative wood protection systems by means of oil treatment. J. Mater. Sci..

[25] Raji O., Tang J.D., Telmadarrehei T., Jeremic D. (2018). Termiticidal activity of chitosan against the subterranean termites *Reticulitermes flavipes* and *Reticulitermes virginicus*. Pest. Manag. Sci..

[26] Telmadarrehei T., Tang D.J., Raji O., Rezazadeh A., Jeremic D. Effect of chitosan on diversity and number of protists in subterranean termites. Proceedings of the 114th Annual Meeting of the American Wood Protection.

[27] Messenger M.T. (2004). The Termite Species of Louisiana: An Identification Guide.

[28] Foster B.T., Cognato A.I., Gold R.E. (2004). DNA-based identification of the eastern subterranean termite, *Reticulitermes flavipes* (Isoptera: Rhinotermitidae). J. Econ. Entomol..

[29] Altschul S.F., Gish W., Miller W., Myers E.W., Lipman D.J. (1990). Basic local alignment search tool. J. Mol. Biol..

[30] Klindworth A., Pruesse E., Schweer J.P., Quast C., Horn M., Glokner F.O. (2013). Evaluation of general 16S ribosomal RNA gene PCR primers for classical and next-generation sequencing-based diversity studies. Nuclei. Acids Res..

[31] Caporaso J.G., Lauber C.L., Costello E.K., Berg-Lyons D., Gonzalez A., Stombaugh J., Knights D., Gajer P., Ravel J., Fierer N. (2011). Moving pictures of the human microbiome. Genome Biol..

[32] Callahan B.J., McMurdie P.J., Rosen M.J., Han A.W., Johnson A.J.A., Holmes S.P. (2016). DADA2: High-resolution sample inference from Illumina amplicon data. Nat. Methods.

[33] Faith D.P., Baker A.M. (2006). Phylogenetic diversity (PD) and biodiversity conservation: Some bioinformatics challenges. Evol. Bioinformat..

[34] Pielou E.C. (1966). The measurement of diversity in different types of biological collections. J. Theor. Biol..

[35] Anderson M.J., Crist T.O., Chase J.M., Vellend M., Inouye B.D., Freestone A.L., Sanders N.J., Cornell H.V., Comita L.S., Davies K.F. (2011). Navigating the multiple meanings of β diversity: A roadmap for the practicing ecologist. Ecol. Lett..

[36] Mikaelyan A., Köhler T., Lampert N., Rohland J., Boga H., Meuser K., Brune A. (2015). Classifying the bacterial gut microbiota of termites and cockroaches: A curated phylogenetic reference database (DictDb). Syst. Appl. Microbiol..

[37] Weiss S., Xu Z.Z., Peddada S., Amir A., Bittinger K., Gonzalez A., Lozupone C., Zaneveld J.R., Vázquez-Baeza Y., Birmingham A. (2017). Normalization and microbial differential abundance strategies depend upon data characteristics. Microbiome.

[38] Badri M., Kurtz Z., Muller C., Bonneau R. (2018). Normalization methods for microbial abundance data strongly affect correlation estimates. bioRxiv..

[39] Zhao Y., Park R.D., Muzzarelli R.A. (2010). Chitin deacetylases: Properties and applications. Mar. Drugs.

[40] Sandoval-Mojica A.F., Scharf M.E. (2016). Gut genes associated with the peritrophic matrix in *Reticulitermes flavipes* (Blattodea: Rhinotermitidae): Identification and characterization. Arch. Insect Biochem. Physiol..

[41] Iida T., Ohkuma M., Ohtoko K., Kudo T. (2000). Symbiotic spirochetes in the termite hindgut: Phylogenetic identification of ectosymbiotic spirochetes of oxymonad protists. FEMS Microbiol. Ecol..

[42] Stingl U., Radek R., Yang H., Brune A. (2005). “Endomicrobia”: Cytoplasmic symbionts of termite gut protozoa form a separate phylum of prokaryotes. Appl. Environ. Microbiol..

[43] Hongoh Y., Sato T., Noda S., Ui S., Kudo T., Ohkuma M. (2007). *Candidatus* Symbiothrix dinenymphae: Bristle-like Bacteroidales ectosymbionts of termite gut protists. Environ. Microbiol..

[44] Borges F.P., Gottardi B., Stuepp C., Larré A.B., de Brum Vieira P., Tasca T., De Carli G.A. (2007). Morphological aspects of *Monocercomonas* sp. and investigation on probable pseudocysts occurrence. Parasitol. Res..

[45] Boykin M.S., Stockert L., Buhse H.E., Smith-Somerville H.E. (1986). *Trichomitus trypanoides* (Trichomonadida) from the termite *Reticulitermes flavipes*. II. Fine structure and identification of the cloned flagellate. Trans. Am. Microsc. Soc..

[46] Tai V., James E.R., Nalepa C.A., Scheffrahn R.H., Perlman S.J., Keeling P.J. (2015). The role of host phylogeny varies in shaping microbial diversity in the hindguts of lower termites. Appl. Environ. Microbiol..

[47] Berlanga M., Palau M., Guerrero R. (2018). Gut microbiota dynamics and functionality in *Reticulitermes grassei* after a 7-day dietary shift and ciprofloxacin treatment. PLoS ONE.

[48] Janzow M.P., Judd T.M. (2015). The termite *Reticulitermes flavipes* (Rhinotermitidae: Isoptera) can acquire micronutrients from soil. Environ. Entomol..

[49] Waidele L., Korb J., Voolstra C.R., Dedeine F., Staubach F. (2019). Ecological specificity of the metagenome in a set of lower termite species supports contribution of the microbiome to adaptation of the host. Anim. Microbiome.

[50] Tang J.D., Raji O., Peterson D.G., Jeremic-Nikolic D. Dysbiosis: A potential novel control strategy for control of subterranean termites. Proceedings of the 114th Annual Meeting of the American Wood Protection Association.

[51] Peterson B.F., Stewart H.L., Scharf M.E. (2015). Quantification of symbiotic contributions to lower termite lignocellulose digestion using antimicrobial treatments. Insect Biochem. Mol. Biol..

[52] Raji O., Tang J.D., Telmadarrehei T., Jeremic D. Analysis of hindgut bacterial phyla frequency and diversity in subterranean termites exposed to chitosan-treated wood. Proceedings of the IRG48 Scientific Conference on Wood Protection.

[53] Su L., Yang L., Huang S., Li Y., Su X., Wang F., Bo C., Wang E.T., Song A. (2017). Variation in the gut microbiota of termites (*Tsaitermes ampliceps*) against different diets. Appl. Biochem. Biotechnol..

[54] Tokuda G., Mikaelyan A., Fukui C., Matsuura Y., Watanabe H., Fujishima M., Brune A. (2018). Fiber-associated spirochetes are major agents of hemicellulose degradation in the hindgut of wood-feeding higher termites. Proc. Natl. Acad. Sci. USA.

[55] Hu H., da Costa R.R., Pilgaard B., Schiøtt M., Lange L., Poulsen M. (2019). Fungiculture in termites is associated with a mycolytic gut bacterial community. Msphere.

[56] Uchida Y. (1988). Antibacterial activity of chitin and chitosan. Food Chem..

